# Expediting pathogen genomics adoption for enhanced foodborne disease surveillance in Africa

**DOI:** 10.1016/j.ebiom.2024.105500

**Published:** 2024-12-18

**Authors:** Aquillah M. Kanzi, Stella I. Smith, Chisomo Msefula, John Mwaba, Abraham Ajayi, Geoffrey Kwenda, Collins K. Tanui, Anthony M. Smith, Linda A. Bester, Firehiwot A. Derra, Kaunda Yamba, Daniel L. Banda, John B. Kalule, Happiness H. Kumburu, Yasmina J. Fakim, Nyasha Sithole, Patrick M.K. Njage, Francis F. Chikuse, Pascale Ondoa, Sofonias K. Tessema, Ebenezer Foster-Nyarko

**Affiliations:** a African Society for Laboratory Medicine, Johannesburg, South Africa; b School of Laboratory Medicine and Medical Sciences, College of Health Sciences, University of KwaZulu Natal, South Africa; c Molecular Biology and Biotechnology Department, Nigerian Institute of Medical Research, Yaba, Lagos, Nigeria; d Pathology Department, Kamuzu University of Health Sciences, Blantyre, Malawi; e Department of Pathology and Microbiology, University Teaching Hospital, Lusaka, Zambia; f Institute of Basic and Biomedical Sciences, Levy Mwanawasa Medical University, Zambia; g Department of Biomedical Sciences, School of Health Sciences, University of Zambia, Lusaka, Zambia; h Africa Centres for Disease Control and Prevention, African Union, Addis Ababa, Ethiopia; i Centre for Enteric Diseases, Division of the National Health Laboratory Service, National Institute for Communicable Diseases, Johannesburg, South Africa; j Department of Medical Microbiology, School of Medicine, Faculty of Health Sciences, University of Pretoria, Pretoria, South Africa; k Biomedical Resource Unit, School of Laboratory Medicine and Medical Sciences, College of Health Sciences, University of KwaZulu Natal, South Africa; l Department of Biochemistry and Microbiology, Faculty of Science, Engineering and Agriculture, University of Venda, South Africa; m Food Safety and Food Microbiology National Reference Laboratory, Food Science and Nutrition Research Directorate, Ethiopian Public Health Institute, Ethiopia; n University Teaching Hospitals, Zambia; o Zambia National Public Health Institute, Zambia; p Department of Medical Laboratory Sciences, School of Life Sciences & Allied Health Professions, Kamuzu University of Health Sciences, Malawi; q Makerere University, College of Veterinary Medicine Animal Resources and Biosecurity (CoVAB), Biotechnical and Diagnostic Sciences, Uganda; r Kilimanjaro Clinical Research Institute, Tanzania; s Kilimanjaro Christian Medical Centre, Tanzania; t Kilimanjaro Christian Medical University College, Tanzania; u University of Mauritius, Mauritius; v Research Group for Genomic Epidemiology, National Food Institute, Technical University of Denmark, Denmark; w Department of Infection Biology, London School of Hygiene and Tropical Medicine, London, United Kingdom

**Keywords:** Food-borne diseases, Pathogen genomics, Genomic surveillance, Public health, Whole-genome sequencing, Africa

## Abstract

The role of genomics in public health surveillance has been accentuated by its crucial contributions during the COVID-19 pandemic, demonstrating its potential in addressing global disease outbreaks. While Africa has made strides in expanding multi-pathogen genomic surveillance, the integration into foodborne disease (FBD) surveillance remains nascent. Here we highlight the critical components to strengthen and scale-up the integration of whole genome sequencing (WGS) in foodborne disease surveillance across the continent. We discuss priority use-cases for FBD, and strategies for the implementation. We also highlight the major challenges such as data management, policy and regulatory frameworks, stakeholder engagement, the need for multidisciplinary collaborations and the importance of robust monitoring and evaluation, aiming to bolster Africa's preparedness and response to future health threats.

**Search strategy and selection criteria**

The search strategy employed to inform this manuscript was designed to encompass the breadth and depth of information required for an evidence-based discussion on the integration of genomics into foodborne disease surveillance in Africa. We systematically reviewed published literature from peer-reviewed journals, technical reports, and policy documents from reputable organisations such as the World Health Organization (WHO), Africa Centres for Disease Control and Prevention (Africa CDC), and other global and regional health bodies.

Search terms included but were not limited to: “foodborne diseases in Africa,” “whole genome sequencing,” “genomic surveillance,” “food safety,” “pathogen genomics,” “AMR surveillance,” “Africa Pathogen Genomics Initiative,” and “quality management systems in genomics.” Boolean operators were used to refine search results, and filters for publication years (2000–2024) and languages (English) were applied. Data sources included PubMed, Scopus, Google Scholar, and institutional repositories, alongside consultations with subject-matter experts and unpublished reports.

Studies were selected based on their relevance to the following themes: Epidemiological burden of foodborne diseases in Africa, the utility of genomic tools in public health surveillance, barriers to and facilitators of the implementation of WGS in low- and middle-income countries (LMICs), case studies of genomic surveillance for foodborne pathogens in Africa and other regions and recommendations for infrastructure, workforce, and policy development related to genomics in foodborne disease surveillance. The search also considered the contextual realities of African health systems, prioritising studies that highlighted challenges, opportunities, and solutions specific to LMICs in the African context.

## Introduction

Africa is a diverse continent with a wide range of food sources that vary according to region and climate. The diversity of food sources presents a significant challenge to implementing and enforcing food safety standards due to limited resources and inadequate water, sanitation, and hygiene (WASH) practices. As such, foodborne disease (FBD) remains a major public health concern, with the continent bearing the highest per capita burden of FBD globally.^1^^,^^2^

About 31 foodborne hazards were identified by the World Health Organization (WHO) to have caused an estimated 600 million foodborne illnesses and 420,000 deaths in 2010, based on estimates from the WHO's Foodborne Disease Burden Epidemiology Reference Group. Most (98%) of these foodborne hazards are biological, with diarrhoeal agents such as non-typhoidal *Salmonella*, *Campylobacter* spp. and *Salmonella* Typhi being the most frequent, along with other agents like *Taenia solium* and hepatitis A virus.^2^ However, because research and disease surveillance data from Africa are limited, previous burden estimates are subject to uncertainty.

Conventional molecular typing methods such as Pulsed Field Gel Electrophoresis and Multiple-locus Variable number tandem repeat Analysis have been used to identify and track foodborne illnesses and outbreaks for over two decades. While these techniques have been instrumental in foodborne disease surveillance, they are fraught with limitations such as low discriminatory power, throughput, and inflexibility that hampers their adoption for routine public health. Whole genome sequencing (WGS) overcomes these limitations. Consequently, most public health surveillance platforms have transitioned to WGS for foodborne pathogen surveillance, coupled with integrating new approaches such as machine learning to build more robust foodborne disease surveillance systems.^3^^,^^4^ Thus, integrating WGS into FBD surveillance systems is a compelling force to reckon with in pursuing enhanced disease monitoring and control mechanisms in Africa.

The Africa Pathogen Genomic Initiative (Africa PGI) of the Africa Centres for Disease Control and Prevention (Africa CDC) was launched in 2020 to strengthen multi-pathogen sequencing and bioinformatics capacity of emerging and re-emerging diseases and genomic surveillance of pathogens of public health importance on the continent. Over the past three years, the COVID-19 pandemic has highlighted the added value of WGS to pathogen surveillance, particularly in sub-Saharan Africa, where much of the sequencing data represents pioneering efforts at pathogen genomics. There is now an unparalleled opportunity to harness the power of genomics to address other priority use cases on the African continent besides COVID-19, such as AMR, vaccine preventable disease, malaria, and FBDs.^5^^,^^6^

Consequently, Africa PGI has identified FBD genomics surveillance in Africa as one of the use cases that can systematically add value for public health decision-making in line with the WHO Integrated Disease Surveillance Response (IDSR) programme.^7^ In April 2022, Africa PGI established an Africa-led technical focus group of experts on foodborne diseases to identify priorities, map key stakeholders and develop a roadmap and implementation strategy for systematic and stepwise integration of genomics for high-priority FBDs in Africa. This report emanates from the work of the FBD focus group and outlines a comprehensive end-to-end framework for integrating genomic surveillance into existing surveillance systems. It provides a clear roadmap and use cases to guide member states, regions, and the continent in establishing, enhancing, and scaling up of genomic surveillance for foodborne disease that is tailored to the local contexts and needs.

## Accelerating genomic surveillance for priority foodborne pathogens

While genomics holds immense potential for revolutionising foodborne disease surveillance, its adoption in low- and middle-income countries (LMICs), notably in most African states, faces significant hurdles.^8^ These challenges span from underdeveloped public health infrastructure, weak surveillance systems, and inadequate sampling techniques to more systemic issues like inefficient supply chain, elevated sequencing operational costs, and limited resources to support FBD genomics surveillance and outbreak investigation. Additionally, there's an evident gap in the skilled workforce, data management capabilities, regulatory policy frameworks, and standardisation of methods.^8^

**Fig. 1:**
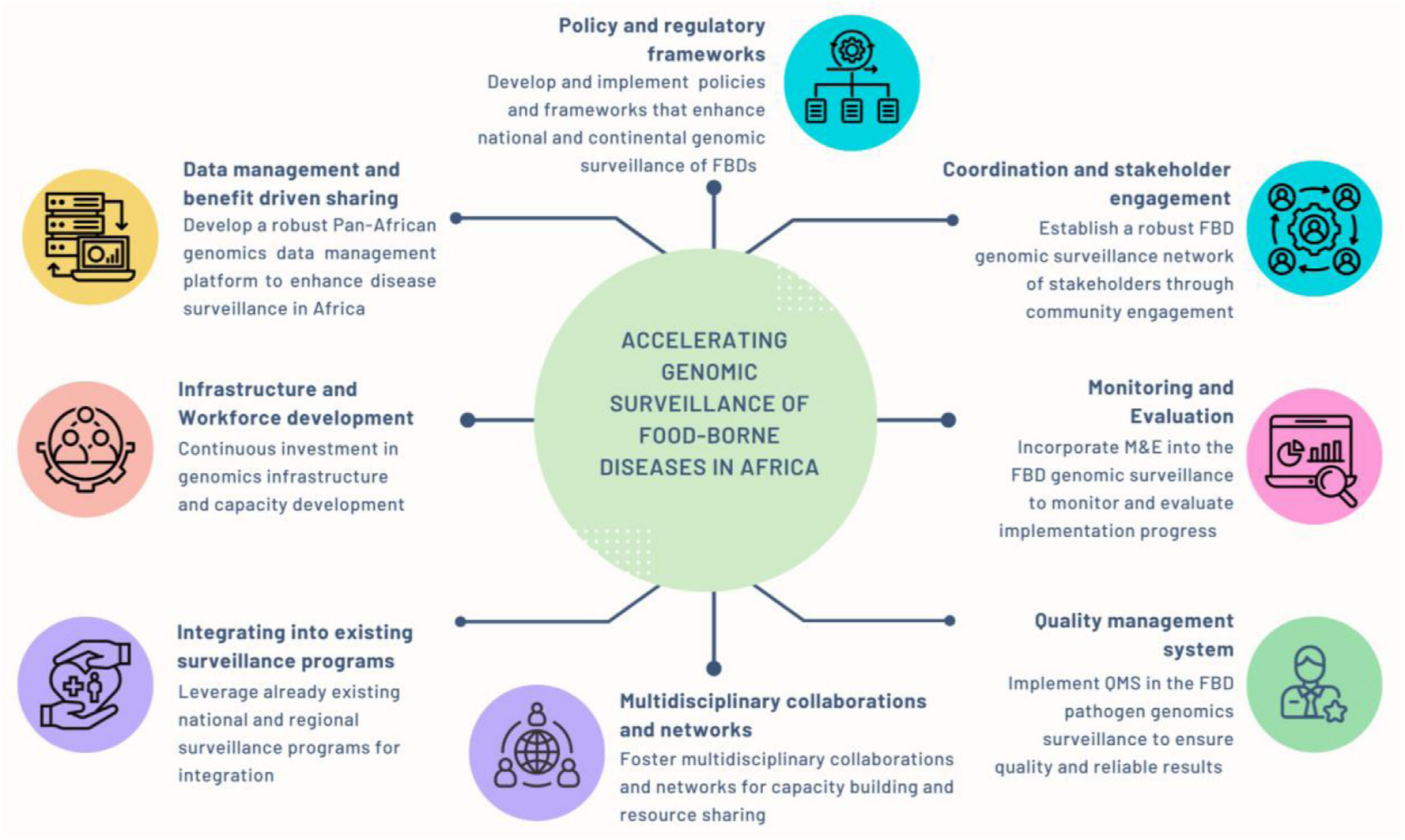
Key elements for the acceleration genomic surveillance for foodborne diseases in African Public Health.

Recognising these challenges, the Africa PGI has taken a proactive approach in underscoring the need for a comprehensive strategy to enhance the utility of genomics in public health and counter the unique challenges posed by LMICs. This approach delineates the critical elements for action, involving a synergistic blend of capacity building, workforce development, policies, collaborations, rigorous quality management, and data systems (Fig. 1).

## Prioritisation of FBD use cases for genomic surveillance in Africa

Considering the growing capacity of genomics in Africa, prioritising foodborne diseases for genomic surveillance is necessary to ensure feasibility, cost effectiveness, and value addition that can safeguard sustainability in the long term. Priority genomic use-cases for FBDs were identified based on data from WHO's FBD Epidemiology Reference Group. The WHO's FDB Epidemiology Reference Group estimated the highest burden of FBD to occur in Western, Central, Eastern and Southern Africa, exceeding 1200 Disability Adjusted Life Years per 100,000 population.^1^^,^^2^ Most (91%) of this mortality is attributed to diarrhoeal agents. Of this, 64% are of bacterial origin, with non-typhoidal *S. enterica*, enterotoxigenic *Escherichia coli* (ETEC), enteropathogenic *E. coli* (EPEC), *Shigella* spp., *Vibrio cholerae* and *Campylobacter* spp*.* among the top aetiological agents.^1^^,^^2^

Growing evidence has emphasised the importance of *Listeria* spp. as a causative agent for FBD in Africa. *Listeria* spp., particularly *L. monocytogenes*, has been isolated from various food, animal, and environmental sources in countries such as Nigeria, South Africa, Ghana, Ethiopia, Egypt, and Botswana.^9^ The 2017/2018 South African listeriosis outbreak brought to global attention the potential for unrecognized, large-scale listeriosis epidemics throughout Africa.^10^

Consequently, non-typhoidal *Salmonella*, *E. coli* and *Shigella* species, *Campylobacter* spp. and *L. monocytogenes* were identified as priority FBD use cases for regional genomic surveillance. While the list of priority pathogens applies to most African countries, each country must conduct a comprehensive prioritisation process to map priority pathogens that are most applicable to their setting, capacity, and capabilities.

## Integration into existing national surveillance systems

International disease control efforts have highlighted the utility of genomics-based surveillance in rapidly detecting outbreaks, resolving source attribution, and tracking the spread of pathogens and AMR—owing to the high resolution afforded by high-throughput sequencing technologies, thus enabling fine-grain microbial genotyping and the resolution of outbreak and transmission clusters.^11^

The path to integrating WGS for foodborne disease surveillance into national surveillance platforms is critical and will require national strategies and regional coordination. The global response to the COVID-19 pandemic increased investments in genomics for public health surveillance.^12^ Presently, more than 36 out of the 55 member states of the African Union have sequencing capacities and capabilities, at least for SARS-CoV-2, in their public health laboratories.^12^^,^^13^ Multiple and varied sequencing platforms are available for use continent-wide through laboratory networks. In addition, the selected use case pathogens for FBD surveillance are among the priority pathogens for WHO integrated disease surveillance and response (IDSR).^7^ However, successfully integrating and implementing WGS for FBD surveillance will require increased coordination across sectors and country-specific estimates for allocation of resources including financial budget to support requisite activities.^11^

**Fig. 2:**
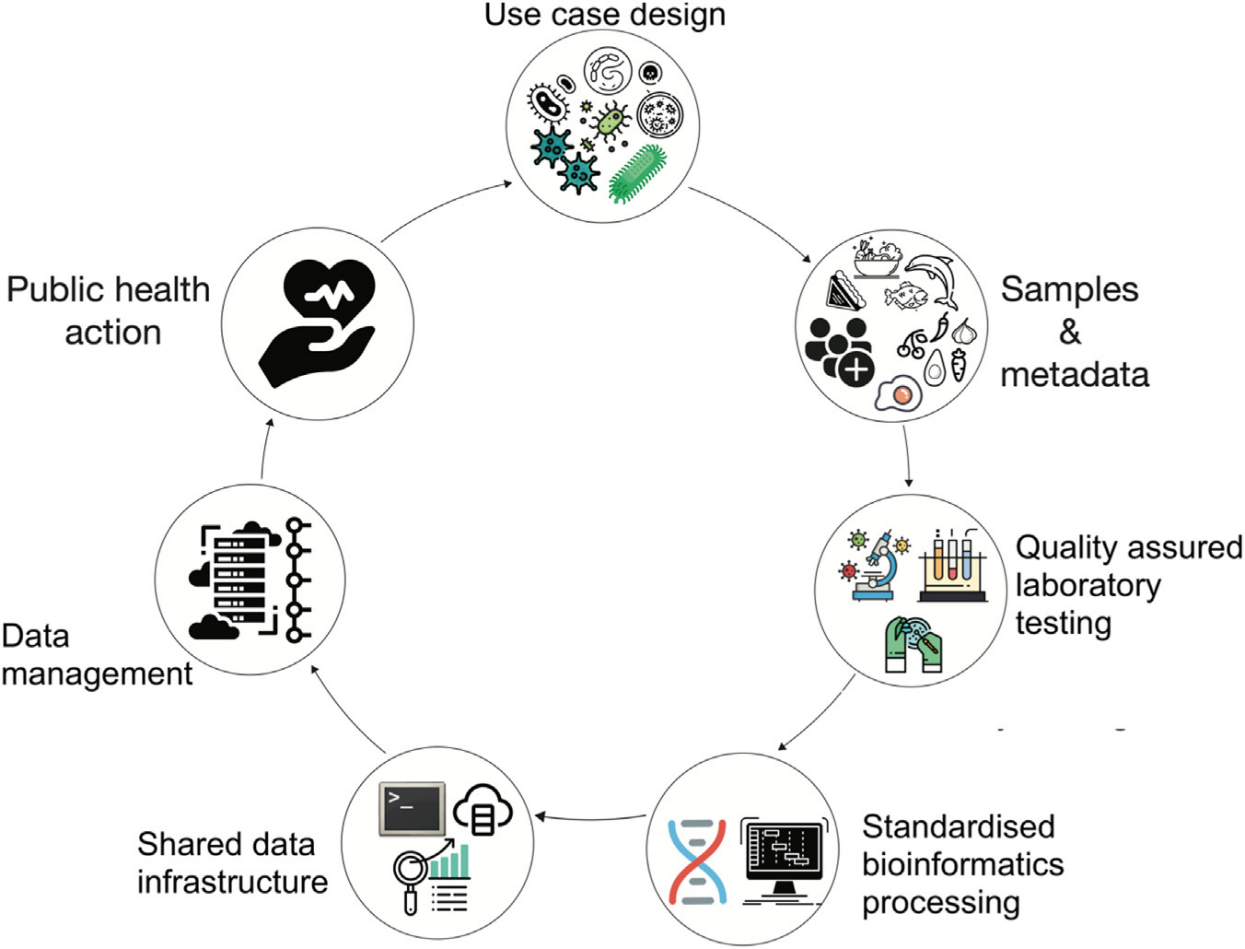
An end-to-end design for implementing FBD genomic surveillance in Africa.

**Fig. 3:**
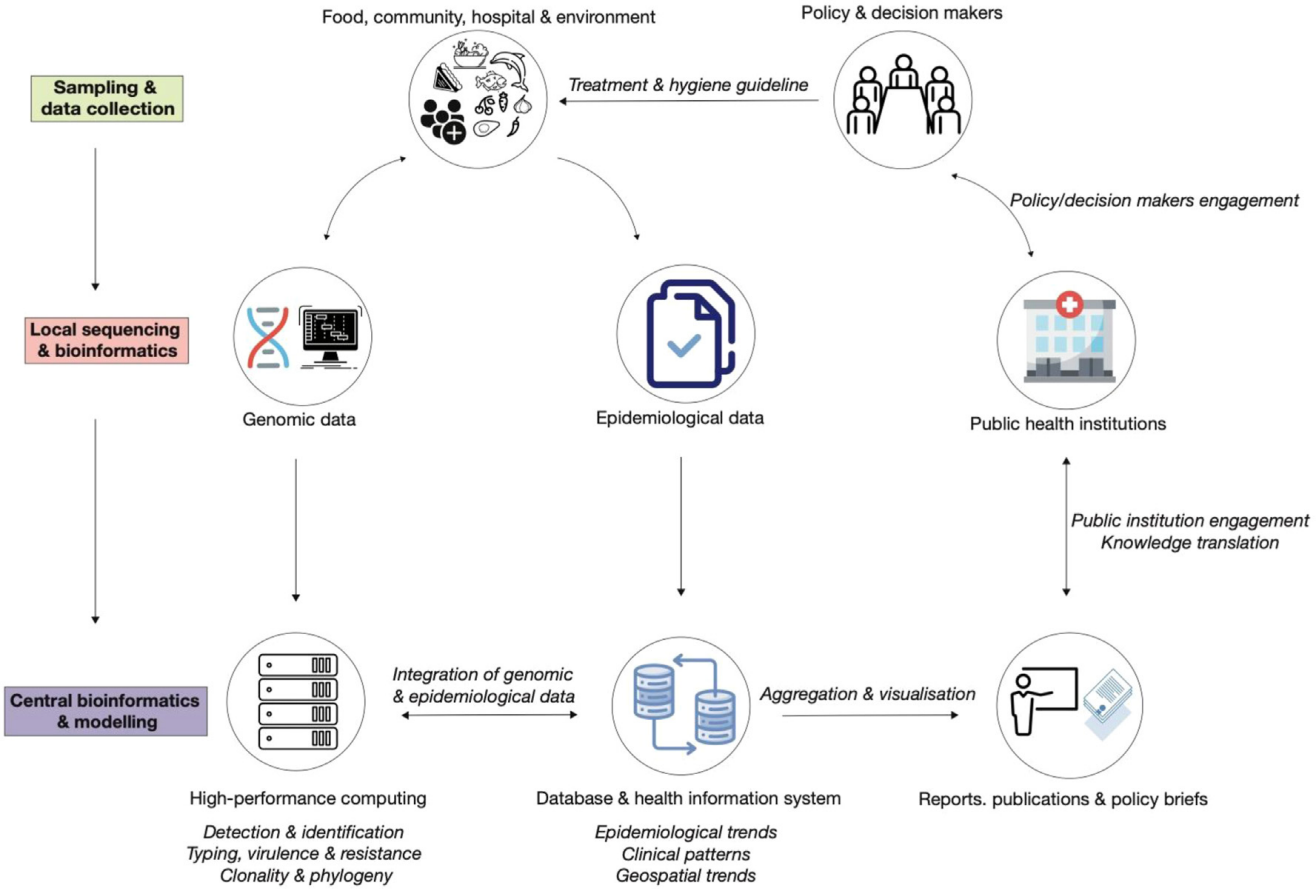
The cross-sectorial unifying elements of national and regional FBD genomic surveillance.

We propose an end-to-end design for implementing WGS for FBD surveillance in Africa, from use case design to public health action (Fig. 2). WGS is intrinsically a cross-sectoral unifying factor involving the multidisciplinary collaboration of various professionals to understand the origin, spread, transmission, and evolution of infectious diseases at the molecular level,^4^ (Fig. 3). Pilot surveillance projects generating and integrating data from multiple sources, including the environment, farms, processing plants, retail outlets, and hospitals, will be necessary as a proof of concept to win political support for investment at the national, regional, and continental levels.

Subsequently, countries must evaluate their genomic surveillance capabilities to determine the appropriate genomic surveillance activities can be successfully implemented. Some surveillance activities could be outsourced to well-resourced regional reference laboratories based on within-country capacities and the findings from pilot projects.^8^ In this regard, the continental pathogen genomics laboratory network is vital in supporting nations in the comprehensive process of end-to-end implementation of WGS for FBD surveillance.

## Investing in infrastructure and workforce development

The complexities of genomic sequencing and data analysis demand specialised skills, which remain scarce in Africa. This, coupled with the fiscal constraints creates barriers to the growth and expansion of cutting-edge genomic laboratories in public health.

Investing in infrastructure, workforce development, and retention is pivotal for advancing genomics in Africa and requires a concerted effort from governments, international organisations, academia, public health agencies, and other stakeholders. Furthermore, robust regional collaborations are vital in facilitating technology transfers, paving the way for a more unified and skilled genomics landscape in Africa.

The Africa CDC Framework for Public Health Workforce Development, 2020–2025,^14^ underscores the need to amplify capacity in public health across African Union member states, particularly in epidemiology and laboratory practice. WGS workflows are intricate and multifaceted, requiring sophisticated analysis infrastructure and significant financial investments twinned with a highly specialised workforce.

Through the Africa PGI, the Africa CDC has already demonstrated its commitment by equipping over 36 member states with genome sequencing tools, essential reagents, ancillaries, and crucial short-term training in both wet and dry laboratory environments. Moreover, short-term bioinformatics training fellowships are currently available for public health professionals to bridge the knowledge gap temporarily. Expanding tailored training programs to resonate with Africa's unique challenges and needs is the cornerstone to nurturing a new generation skilled in foodborne pathogen genomics.^15^

## Implementing quality management systems for pathogen genomics

Implementing quality management systems (QMS) in genomics laboratories presents notable hurdles. WGS workflows are intricate and multifaceted, necessitating careful navigation to ensure accurate and reproducible results. Additionally, QMS for bioinformatics are currently under development.^16^ Quality standards often implemented in clinical and public health laboratories, such as ISO 15189 and ISO 17025, may not effectively address quality issues arising from WGS integration. Implementing ISO standards requires significant capital investment, which can further strain already limited public health funds. If these gaps in quality management are not sufficiently addressed, they could impact the quality of test results and surveillance outcomes, thereby affecting public health actions. The Centres for Disease Control and Prevention (CDC) NGS Quality Workgroup has developed NGS-focused QMS based on the 12 Quality Systems Essentials (QSEs) defined by the Clinical & Laboratory Standards Institute (CLSI).^17^ Additionally, ISO 23418 has recently been established to guide the WGS of bacteria obtained from the food chain.^18^ While these developments of QMS around WGS are a significant step, they may need to be customised to address the unique challenges in Africa. This presents an opportunity for the Africa CDC, in collaboration with organisations such as the African Society for Laboratory Medicine (ASLM) and Public Health Alliance for Genomic Epidemiology (PHA4GE), to develop guidelines for the effective adoption and implementation of NGS-focused QMS in Africa.

## Data management and benefit-driven sharing

The COVID-19 pandemic highlighted numerous challenges related to sharing genomic data, including issues of trust, benefit distribution, data protection, intellectual property, and ethical considerations. For example, the sharing of the Omicron (B.1.1.52a) variant by Southern African nations led to unexpected, disheartening social and economic repercussions. Notwithstanding, the value of genomic data-sharing is evident; it has improved the speed and effectiveness of responses to public health threats, from disease outbreaks to foodborne illnesses.

While global data-sharing platforms like the NCBI Pathogen Detection system have facilitated significant contributions from African foodborne pathogen sequences, there remains a critical need for a dedicated, centralised platform tailored to the unique challenges of foodborne diseases in Africa. Such a platform would enable local integration, quality control, and analysis of pathogen sequences, allowing for the timely detection of potential clusters in collaboration with epidemiologists. Additionally, it would complement global repositories by streamlining sequence deposition, following models used by international foodborne surveillance systems, such as PulseNet USA.^19^

**Fig. 4:**
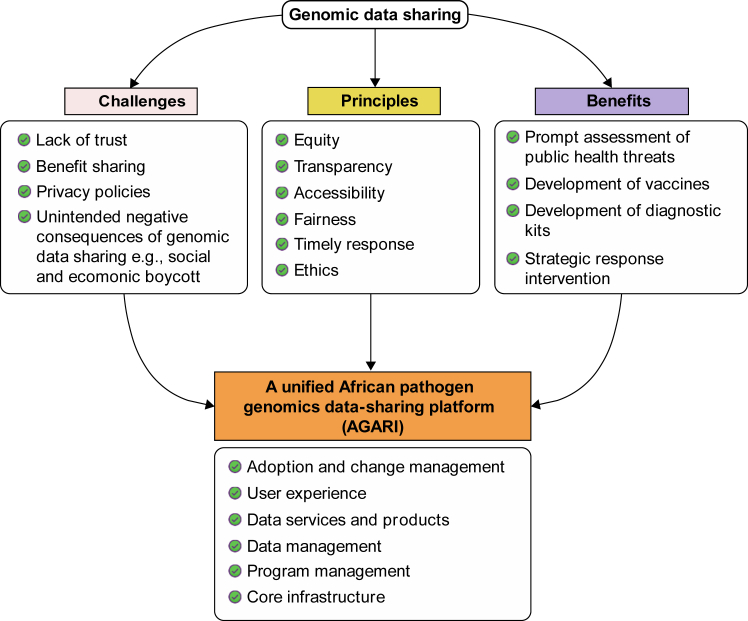
The holistic approach hinged on six pillars for developing a pan-African pathogen genomics data-sharing platform (AGARI) [apaportal.sanbi.ac.za].

Ongoing efforts to develop an African data platform should be supported and accelerated, ensuring that stakeholders can not only deposit data into existing global repositories but also effectively integrate and analyse it for public health action. Incorporating robust data management and sharing strategies into genomic surveillance of foodborne diseases in Africa is crucial. The efforts of the Africa CDC and its collaborators have been noteworthy, enabling Africa to produce genomic data during the pandemic. However, a unified and structured data management system is essential to sustain this progress. This is particularly timely as the Africa PGI, supported by the Africa CDC, advances in outlining strategies for incorporating genomics into pathogen surveillance across the continent. Christoffels et al., (2023)^20^^,^^21^ stress the importance of a centralised African pathogen genomics data-sharing platform (AGARI), which considers best practices, potential obstacles, and benefits (Fig. 4). Bedeker et al., (2022)^22^ showcase the advantages of creating a structured approach with tools to guide stakeholders in recognising and leveraging both public databases and private data-sharing opportunities.

## Policy and regulatory frameworks

To bolster the genomic surveillance of foodborne pathogens in Africa, it is imperative to establish comprehensive policy and regulatory frameworks (PRFs) to address the challenges outlined earlier. Regrettably, many African nations currently lack these PRFs. National PRFs should ensure protection of data privacy to uphold the dignity, autonomy, and confidentiality of data (whether it is from human subjects or pathogens) and avert potential discrimination, stigmatisation, or exploitation based on genetic insights.^23^^,^^24^ Data privacy policies and regulations should be robust to comprehensively inform genomic data sharing and intellectual property rights policies that champion responsible and secure genomic data dissemination balancing the rights and interests of data providers and users, ensuring equitable recognition, attribution, and benefit-sharing. PRFs should also outline ethical guidelines on responsible collection, utilisation, and storage of genomic data in alignment with prevailing national, regional, and international ethical standards. In addition, delineating roles and responsibilities for all stakeholders—including researchers, laboratories, health authorities, and data custodians—is necessary.^20^

Merely crafting PRFs isn't sufficient. Successful implementation hinges on robust governance and open communication channels among all stakeholders. These entities are pivotal in seamlessly integrating genomics into foodborne disease surveillance initiatives.

## Coordination and stakeholder engagement

The Africa CDC through the Africa PGI can bring together public health laboratories into a cohesive FBD surveillance network. Drawing insights from the triumphs and hurdles encountered by PulseNet Africa in orchestrating a continent-spanning WGS for FBD surveillance can be beneficial.^25^ A pivotal step in this process is conducting a detailed assessment of public health laboratories using standardised tools. This will ascertain the level of national governmental support, pinpoint workforce gaps, evaluate sequencing infrastructure needs, identify data gaps, and check the compatibility of standard operating procedures. This audit's findings will be instrumental in shaping a framework that optimises limited resources, ensuring the consistent involvement of all 55 African nations in the surveillance network. The devised framework will transparently designate eligible laboratories as reference facilities, supporting labs needing sustained capacity enhancement.

Africa PGI should act as a linchpin, aligning the efforts of NPHIs with academic institutions and research entities, by optimising and leveraging capacities to enhance FBD genomic surveillance in Africa.^8^ Engaging the broader community is paramount for detecting, investigating, and reporting FBD outbreaks. It's worth noting that WGS FBD source identification might carry ethical ramifications, potentially leading to community stigmatisation. As such, FBD genomics surveillance efforts should prioritise community outreach, leveraging formal education, community advisory panels, and digital platforms like social media. Valuable insights can be gleaned from pioneering, public-centric endeavours like South Africa's and The Gambia's speaking book projects.^26^, ^27^, ^28^

## Multidisciplinary collaborations and networks

We have successfully catalogued key stakeholders, financial supporters, and partners dedicated to the genomic surveillance of FBD priority use cases. Such a comprehensive database is a cornerstone for nurturing collaborative endeavours and establishing networks vital for genomic surveillance across the continent. A sound WGS strategy targeting foodborne diseases in Africa must be instituted, emphasising symbiotic relationships among laboratories, academic and research institutions, and prominent networks like PulseNet Africa, SeqAfrica, and H3ABioNet, along with other related research entities.^26^, ^27^, ^28^ Unfortunately, the current landscape reveals a shortfall in collaboration and inter-networking among various genomic-centric factions in Africa. This disjointed environment curtails initiatives aimed at stimulating discussions on cultivating local analytical capabilities within African nations, including foundational elements like infrastructure, informatics, and data stewardship.^29^

Furthermore, a robust WGS framework necessitates the establishment of critical capacities before implementation. According to WHO guidelines,^30^ a functional event-based surveillance system must exist, capable of detecting foodborne disease outbreaks. Minimum requirements include sufficient epidemiological capacity to investigate outbreaks, well-equipped public health laboratories for pathogen identification, and regulatory frameworks that empower food safety authorities to act based on surveillance data. Many African countries have existing food safety board's responsible for monitoring products on the market and recalling contaminated goods. For example, in South Africa, food safety oversight is managed by the Directorate of Food Control within the Department of Health.^31^ In Nigeria, the National Agency for Food and Drug Administration and Control (NAFDAC) serves as the regulatory body equivalent to the FDA. Ghana's Food and Drugs Authority is similarly tasked with ensuring food safety.

However, significant gaps remain in the rapid identification and notification of outbreaks by national public health laboratories, as well as clarity on what constitutes actionable data for enforcement. To enhance foodborne disease surveillance, we recommend that the isolation of organisms included in the FBD use case priority list—such as *Salmonella*, *E. coli*/*Shigella*, *Campylobacter*, *Vibrio*, and *Listeria*—be flagged as “notifiable” to national reference laboratories upon routine isolation at sentinel laboratories. These laboratories should store these isolates for onward shipment to regional reference laboratories for nucleic acid extraction and sequencing.

We also recommend implementing a system for regular scheduled shipments of the isolates to the regional reference laboratories for molecular and genomic characterisation. Regulatory frameworks must be established to empower these authorities to act swiftly based on surveillance data, ensuring timely interventions to protect public health.

Collaborative dynamics and integrated networking can pave the way to address these intricate challenges, encompassing the legal and ethical facets of genomics data governance and distribution. Elevating the spirit of collaboration becomes paramount, primarily when it facilitates the standardisation of sequencing methodologies employed in genomic data derivation, coupled with synchronising metadata documentation and dissemination across Africa.^12^ Foundational aspects, such as Quality Management Systems (QMS)—pivotal to producing high-quality genomic data—can be seamlessly adopted under robust collaborative and network-driven frameworks.^25^ This communal approach resonates with the tenets established in the WHO's ‘Defining Collaborative Surveillance’ document,^32^ which outlines core concepts for strengthening health emergency preparedness, response, and resilience.

## Robust monitoring and evaluation plan

To successfully integrate genomics into foodborne pathogen surveillance within Africa, it's paramount to adopt thorough and innovative Monitoring and Evaluation (M&E) methodologies spearheaded by the Africa CDC. An adept M&E strategy serves to unearth potential loopholes while simultaneously revealing avenues for improvement. Key indicators should gauge the reach and efficacy of sequencing facilities; assessing the impact of initiatives focused on bolstering WGS data creation, interpretation, and dissemination within nations and the broader African domain; scrutinising the implementation fidelity of strategic roadmaps post-adoption; and gathering insights from a diverse cohort of stakeholders concerning decision-making paradigms derived from genomic data, especially in the realm of addressing foodborne disease epidemics across the continent.

Furthermore, the critical indicators outlined in the WHO's global strategy for genomic surveillance—particularly for pathogens with heightened pandemic and epidemic susceptibilities—warrant meticulous consideration.^33^^,^^34^

## Conclusions

The application of genomics for public health surveillance, including foodborne diseases, is gaining traction in Africa. Investment in genomics and bioinformatics infrastructure during the COVID-19 pandemic has better positioned African countries regarding pandemic preparedness. However, glaring deficiencies hindering implementation and expansion of use-cases for FBD have become apparent. The key elements discussed in this article could accelerate the implementation of national and regional action plans for genomics surveillance of foodborne diseases for public health action. Implementing priority use cases should be informed by continuous needs and readiness assessment to identify laboratories needing assistance and streamline national, regional, and continental capacity monitoring and evaluation.

### Outstanding questions

Despite significant advancements, several critical challenges remain for the effective implementation and sustainability of genomics-based foodborne disease surveillance in Africa. These include how countries can systematically identify and prioritise pathogens for genomic surveillance while accounting for their unique epidemiological and resource constraints, as well as the development of innovative funding models to ensure the long-term financial sustainability of such systems in resource-limited settings. Additionally, questions remain on how to ensure equitable scalability and access to genomic infrastructure and expertise across Africa Union member states particularly for low-resource regions. What type of frameworks and programs are required that promote trust, equitable data sharing, and community engagement while safeguarding data security, ethical compliance, and benefit-sharing? Furthermore, harmonising policy and regulatory structures to support ethical genomic data use and governance is also essential but gaps remain. Evaluating the public health impact of genomic surveillance programs will require robust metrics that must be established. Addressing these gaps will be critical to harnessing the full potential of genomics in advancing foodborne disease prevention and control in Africa.

## Contributors

Manuscript preparation and writing: Aquillah M. Kanzi, Stella I. Smith, Chisomo Msefula, John Mwaba, Abraham Ajayi, Geoffrey Kwenda and Ebenezer Foster-Nyarko.

Review and approval of the final manuscript: Aquillah M. Kanzi, Stella I. Smith, Chisomo Msefula, John Mwaba, Abraham Ajayi, and Geoffrey Kwenda, Collins K. Tanui, Anthony M. Smith, Linda A. Bester, Firehiwot A. Derra, Kaunda Yamba, Daniel L. Banda, John B. Kalule, Happiness H. Kumburu, Yasmina J. Fakim, Nyasha Sithole, Patrick M.K. Njage, Francis F Chikuse, Pascale Ondoa, Sofonias K. Tessema, and Ebenezer Foster-Nyarko.

## Declaration of interests

The authors declare that they have no competing interests.
